# Hemodynamic numerical simulation of aortic arch modular inner branched stent-graft in eight early patients from the first-in-human case series

**DOI:** 10.3389/fcvm.2022.981546

**Published:** 2022-08-30

**Authors:** Yating Zhu, Fen Li, Hongpeng Zhang, Hui Song, Xiaodan Ma, Long Cao, Wenjun Zhang, Wei Guo

**Affiliations:** ^1^Department of Vascular Surgery, First Medical Center of Chinese People's Liberation Army General Hospital, Beijing, China; ^2^College of Mechanical and Vehicle Engineering, Taiyuan University of Technology, Taiyuan, China; ^3^Equipment Department, The Fourth People's Hospital of Taiyuan, Taiyuan, China; ^4^Department of General Surgery, People's Liberation Army No. 983 Hospital, Tianjin, China; ^5^Department of Ultrasonic Diagnosis, People's Liberation Army No. 980 Hospital, Shijiazhuang, China

**Keywords:** aortic arch, thoracic endovascular aortic repair (TEVAR), thoracic stent-graft, inner branched stent-graft, hemodynamics, numerical simulation

## Abstract

**Background:**

The modular inner branched stent-graft (MIBSG) (WeFlow-Arch™) is an emerging device for challenging aortic arch pathologies. Hemodynamic numerical simulation is conducive to predicting long-term outcomes as well as optimizing the stent-graft design.

**Objective:**

This study aims to analyze the hemodynamic characteristics of the MIBSG devices based on numerical simulation analyses.

**Methods:**

From June 2019 to June 2021, MIBSGs were utilized in eight cases. Numerical simulation analyses of branch perfusion and indicators including the time-averaged wall shear stress, oscillatory shear index, and relative residence time were performed.

**Results:**

Lesions involved Zone 1 (*n* = 2), Zone 2 (*n* = 4), and Zone 3 (*n* = 2). Branched stent-grafts were deployed in the innominate artery and left common carotid artery (*n* = 5) or in the innominate artery and left subclavian artery (*n* = 3). The hemodynamic change in common was increased perfusion in the descending aorta and left common carotid artery. Half of the patients had increased cerebral perfusion of 8.7% at most, and the other half of the patients showed a reduction of 5.3% or less. Case 3 was considered to have acquired the greatest improvement in hemodynamic features.

**Conclusion:**

The MIBSG showed improved hemodynamic features in most cases. The design of the MIBSG could be partly modified to acquire better hemodynamic performance.

## What this paper adds

A multicenter clinical trial (GIANT Study) of an emerging modular inner branched stent-graft (WeFlow-Arch™) for challenging aortic arch lesions is in progress in China. This study presents the results of the numerical simulation analyses that verified significant hemodynamic improvement in most of the first-in-human cases and may allow identification of high-risk patients with potential long-term complications who require close follow-up. Hemodynamic numerical simulation should be performed to guide preoperative planning and optimize device designs for complicated endovascular aortic arch reconstructions.

## Introduction

**Figure 1:**
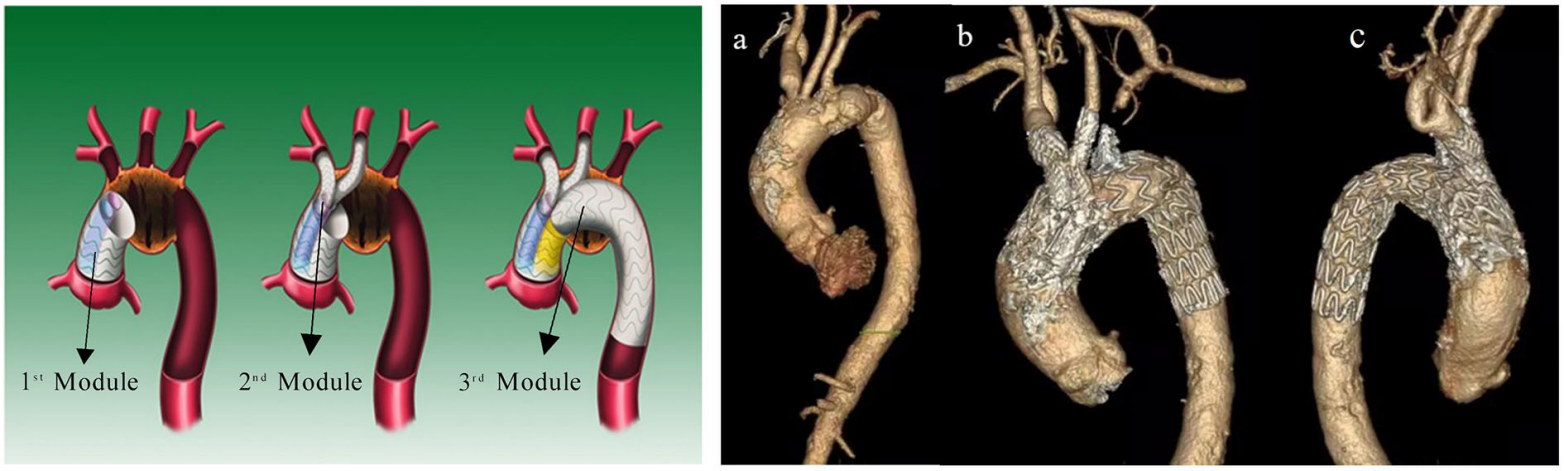
**(Left)** Diagram and **(right)** *in vivo* morphology of MIBSG. First module: main body stent-graft with double embedded tunnels landing in ascending aorta. Second module: two inner bridging stent-grafts to the supra-arch vessels. Third module: distal extending stent-graft in the descending aorta. **(a)** Preoperative morphology of aneurysm. **(b,c)** Postoperative morphology of MIBSG. MIBSG = modular inner branched stent-graft.

Thoracic endovascular aortic repair (TEVAR) for the treatment of aortic arch pathologies (AAPs) involves the creation of a sufficient landing zone with simultaneous branch vessel preservation, making the procedure very challenging. Morphological and physiological factors such as a curved aortic arch, anatomical variations and angles among the branch vessels, and high-speed and high-pressure pulsatile blood flow have significant impacts on the safety and efficacy of endovascular aortic arch reconstruction (1). During the last decade, significant progress has been made in TEVAR for AAPs. The use of stent-grafts (SGs) designed with single or double inner branches has been reported with encouraging early results (2–12). The modular inner branched SG (MIBSG) (WeFlow-Arch™; Weiqiang Medical Technology Co., Ltd., Hangzhou, China) is a contemporary option for the endovascular repair of AAPs. It was first proposed and designed by our center for aortic arch reconstruction in 2005, and the technical feasibility has been verified through animal experiments (13). Based on the promising early postoperative results from the first-in-human cases, a multicenter clinical trial (GIANT Study, NCT04765592, ChiCTR2100044591) is currently in progress in China. In contrast to conventional fenestrated or parallel SGs, the complicated geometry of the MIBSG is characterized by the ascending aorta landing (Zone 0) (14) combined with more metal scaffolding overlaps; these features are considered to have a significant impact on the physiological curvature, elastic deformation, wall stress, and blood flow streamline around the aortic arch (Figure 1). Therefore, a thorough evaluation of the postoperative hemodynamic characteristics and potential risk of late surgery-related complications is necessary.

The current procedural planning and efficacy assessment of TEVAR mainly rely on anatomic criteria of morphological improvement obtained by computed tomography angiography (CTA) with multiplanar reconstruction instead of an in-depth quantitative analysis of hemodynamic characteristics. Patients with good postoperative imaging morphology may still have long-term risks of complications such as endoleaks, stroke, stent collapse or occlusion, or SG-induced new entry (11), indicating that the current treatment strategies and device design should be optimized. In recent years, numerical simulations have been utilized to investigate peri-TEVAR hemodynamic characteristics such as the stress distribution, changes in the flow velocity and flow field, and the friction stability of SGs. Hence, we performed the present fluid dynamics numerical simulation in the early eight patients of the first-in-human MIBSG case series to evaluate the hemodynamic outcomes and accordingly predict the prognosis and optimize the design of the MIBSG device. We obtained written informed consent from every reported patient. The study was approved by the Ethics Committee of the Chinese People's Liberation Army General Hospital (S2018-230-01) and adhered to the principles of the Declaration of Helsinki.

## Materials and methods

### Patients and devices

From June 2019 to June 2021, eight patients with aortic arch aneurysms who underwent interventions with MIBSGs were included in this study. None of the patients had typical chest or back pain, and all had been diagnosed *via* CTA before hospitalization. CTA of the entire aorta was performed at 1 week, 6 months, and 12 months after the intervention and yearly thereafter. The flow velocity of the supra-arch branches was acquired *via* Doppler ultrasound (LOGIQ 9; GE Healthcare, Chicago, IL, USA).

The MIBSG device (WeFlow-Arch™) was manufactured by Weiqiang Medical Technology Co., Ltd. (Hangzhou, China) according to each patient's need. This modular SG consists of three modules (Figure 1). The first module is a cylindrical ascending aortic SG coupled with double embedded tunnels that provide access to the innominate artery (IA) and the left common carotid artery (LCCA) or left subclavian artery (LSA). The second module refers to the branched SGs. The third module is the extension SG in the distal arch and descending aorta (DA). The procedure was performed as described in our previous report (15). Concomitant LCCA–LSA bypass or coil embolization of the LSA was performed if necessary.

### Geometrical reconstructions

**Figure 2:**
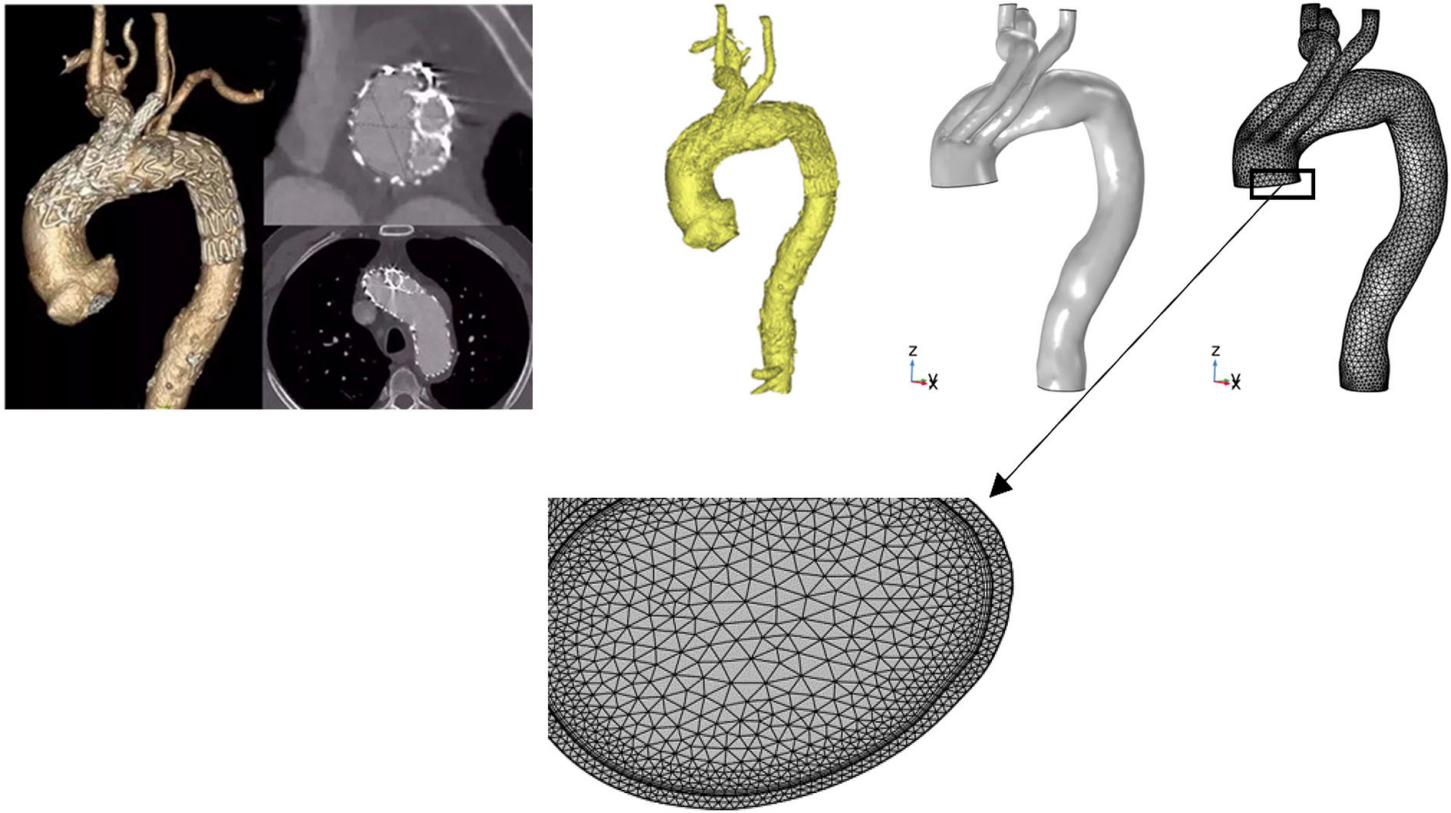
Three-dimensional reconstruction and mesh generation of MIBSG in Case 4. The three-dimensional geometry was reconstructed from computed tomography angiography images using Mimics 19.0. The branches and aortic arch were separated and offset in 3-matic software to obtain the vascular wall. Commercial finite element method software (COMSOL 5) was used for the computation of the hemodynamic flow. To obtain mesh-independent solutions, 2,927,088 elements were used for Case 4. Although the laminar model used in all instances was reportedly able to capture the characteristic flow patterns in the aortic arch, six layers of boundary layer mesh were used to resolve the near-wall flow accurately. MIBSG, modular inner branched stent-graft.

Thin-slice CTA images of all the pathologies were acquired using a 256-row CT scanner (Revolution CT; GE Healthcare) with the following parameters: 512 × 512 × 700; pixel spacing, 0.785/0.785; resolution, 1.274 pixels/mm; and slice thickness, 1 mm. Three-dimensional geometric reconstruction with DICOM-format CTA images was then performed using commercial software (Mimics version 19.0; Materialise NV, Leuven, Belgium). Threshold segmentation and dynamic region growth commands were used to obtain the aortic contour model. The branches and the aortic arch were then separated and offset in 3-matic software (Materialise NV) to obtain the vascular wall with a vessel branch wall thickness of 1.0 mm and aortic wall thickness of 2.0 mm. The final model began above the level of the aortic sinus and ended at the level of the proximal renal artery. Commercial finite element method software (COMSOL 5; COMSOL, Inc., Burlington, MA, USA) was used for the computation of the fluid–structure interaction problem to analyze the coupling effects between blood flow and vessels. In default mode, COMSOL solvers dynamically adjust the time step, and the maximum time step was limited to 0.05 s to ensure that it was fine enough for the achievement of a time step-independent solution (Figure 2).

### Computations framework

The blood flow and corresponding pulsatile vessel deformation substantially involve the fluid–structure interaction issue in computation. Blood is considered a homogeneous and incompressible Newtonian fluid in the aorta. In this study, we describe the blood flow behavior using the incompressible Navier–Stokes equation with the density and viscosity set at 1,060 kg/m^3^ and 0.0035 Pa·s, respectively. An isotropic linear elastic material with Poisson's ratio of 0.49, Young's modulus of 7.5 ×10^5^ Pa, and a density of 1,150 kg/m^3^ was used as the vessel wall. The interaction between the blood and vessel wall was simulated using the arbitrary Lagrangian–Eulerian formulation.

### Boundary condition

**Figure 3:**
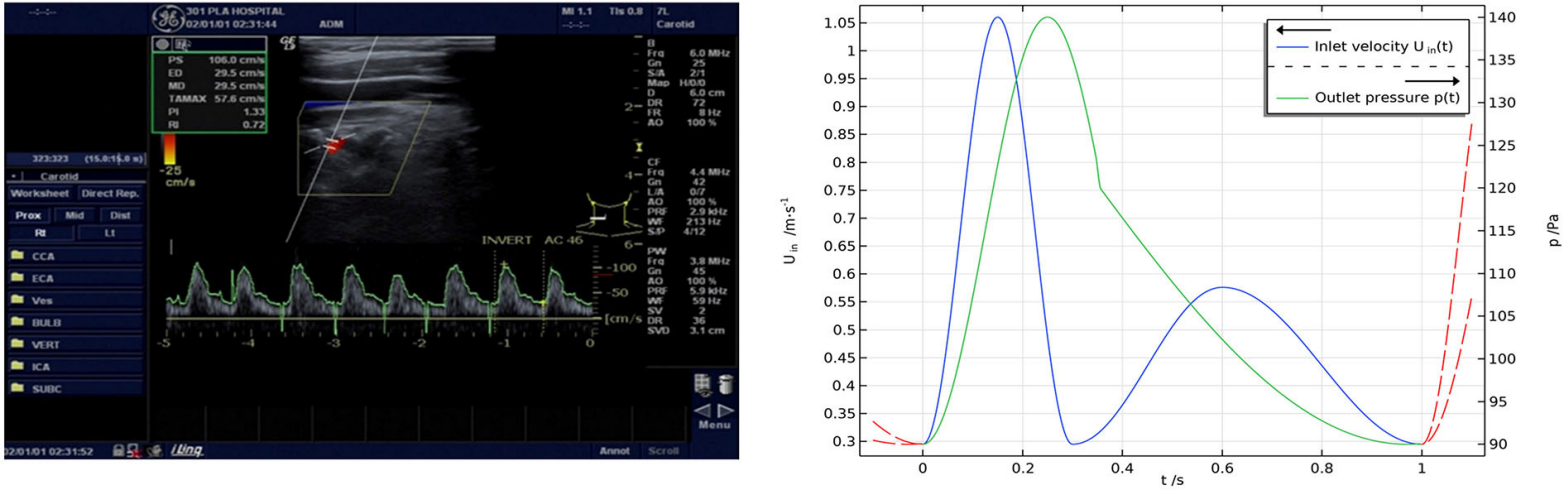
**(Left)** Results of Doppler ultrasonic velocimetry and **(right)** boundary conditions. The inlet boundary condition was set to a velocity condition measured with Doppler ultrasound, and the outlet boundary condition was set to a pressure condition according to clinical monitoring.

The pulsating blood flow within a cardiac cycle was simulated with the velocity boundary condition of the inlet measured with Doppler ultrasound and the pressure boundary condition of the outlet based on clinical monitoring (16, 17) (Figure 3). The formulas for velocity and pressure are as follows:


$$\begin{array}{lll} v\left(t\right) & = & \{\begin{array}{c} -0.3825cos\left(6.6667\pi t\right)+0.6775,\\ 0<t\leq 0.3\;s\\ -0.1405cos\left(3.3333\pi \left(t-0.3\right)\right)+0.4355,\\ 0.3<t\leq 0.6\;s\\ -0.1405cos\left(2.5\pi \left(t-1\right)\right)+0.4355,\\ 0.6<t\leq 1\;s\; \end{array}\\ p\left(t\right) & = & \{\begin{array}{c} -25cos\left(4\pi t\right)+115,\\ 0<t\leq 0.35\;s\\ \left(p\left(0.35\right)-90\right)\cos\left(0.7692\pi \left(t-0.35\right)+\pi -1.5\right)\\ +p\left(0.35\right),\;0.35<t\leq 1\;s \end{array} \end{array}$$

### Analysis of hemodynamic indicators

Three hemodynamic indicators based on wall shear stress (WSS) were quantified and calculated, namely, the time-averaged WSS (TAWSS), oscillatory shear index (OSI), and relative residence time (RRT). TAWSS is a scalar defined as the time-averaged absolute magnitude of the surface traction vector. OSI refers to the wall shear stress oscillations within a cardiac cycle, and RRT represents the residence time of particles in a certain position. Regions with a TAWSS of <0.4 Pa, OSI of >0.25, and RRT of >5/Pa indicate a more remarkable tendency toward atherosclerosis (18). Areas with high-risk regions characterized by abnormal values of these indicators were distinguished to visualize this tendency. According to the mean shear stress defined by Taylor et al. (19) the formulas are expressed as follows:


$$\begin{array}{lll} TAWSS & = & \tau_{abs}=\frac{1}{T}\int_{0}^{T}|\vec{\tau_{w}}|dt\\ \tau_{mean} & = & \left|\frac{1}{T}\int_{0}^{T}\vec{\tau_{w}}dt\right|\\ OSI & = & \frac{1}{2}\left(1-\frac{\tau_{mean}}{\tau_{abs}}\right)\\ RRT & = & \frac{1}{TAWSS\left(1-2\;OSI\right)} \end{array}$$

## Results

### Interventions and morphological features

**Figure 4:**
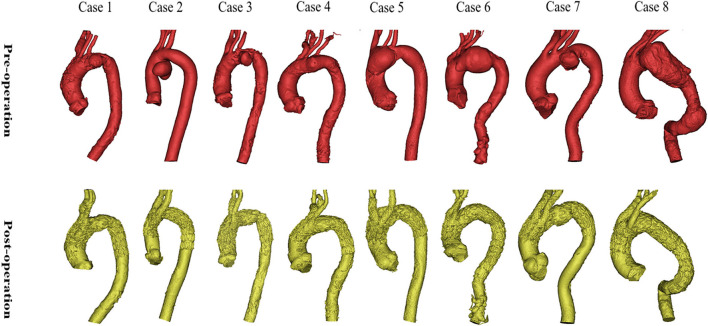
Preoperative and postoperative morphologies of the eight interventions. The morphologies and locations of the eight lesions are shown in the above images. The reconstructions of the arch and branch vessels are shown in the below images.

Details of the eight interventions and morphological features are presented in Table 1 and Figure 4. The diagnoses were saccular aneurysm (*n* = 7) and dissection aneurysm (*n* = 1). The lesions were located in the inner curvature (*n* = 3), anterior wall of the aorta (*n* = 3), or aortic full-cycle (*n* = 2); and involved Zone 1 (*n* = 2), Zone 2 (*n* = 4), or Zone 3 (*n* = 2). The branched SGs were deployed in the IA and LCCA (*n* = 5) or in the IA and LSA (*n* = 3). Concomitant LCCA–LSA bypass was performed in four cases. The original LSA was simultaneously occluded with several detachable fibered coils (Interlock-35; Boston Scientific, Natick, MA, USA). One immediate type I endoleak was observed in Case 5, and the leak had disappeared by the 6-month follow-up CTA.

**Table 1:** Details of the eight interventions.

**Cases**	**Lesions**	**Locations**	**Branch SGs**	**Assistant procedurals**	**Temporary pacemaker utilization**	**Complications**
Case 1	Aneurysm	Zone 3	IA & LCCA	LCCA-LSA bypass, LSA embolization with coils	+	None
Case 2	Aneurysm	Zone 1	IA & LSA	LCCA-LSA bypass, proximal LCCA ligation	+	None
Case 3	Aneurysm	Zone 3	IA & LCCA	None	+	None
Case 4	Aneurysm	Zone 2	IA & LCCA	None	+	None
Case 5	Dissection aneurysm	Zone 1	IA & LSA	LCCA-LSA bypass, proximal LCCA ligation	+	Proximal endoleak (diminished in 6-months CTA)
Case 6	Aneurysm	Zone 2	IA & LCCA	LCCA-LSA bypass, LSA embolization with coils	+	None
Case 7	Aneurysm	Zone 2	IA & LCCA	None	+	None
Case 8	Aneurysm	Zone 2	IA & LSA	None	+	None

+, yes; CTA, computed tomography angiography; IA, innominate artery; LCCA, left common carotid artery; LSA, left subclavian artery; SGs, stent-grafts.

### Numerical simulations

#### Blood perfusion redistribution

The postoperative geometric morphologies of the SGs were good with fluent blood flows. The flow rate of each inlet and outlet was calculated *via* surface integration. Generally, the postoperative perfusions of branches differ from the preoperative baseline situation because of the influence of the MIBSG as well as the embolized LSA. Some changes in common included generally increased perfusions of the DA and LCCA, whereas the perfusion of the IA, right subclavian artery (RSA), and right common carotid artery (RCCA) decreased with some exceptions. The LSA perfusion was not calculated because most of the patients underwent intraoperative LSA coverage or embolization. Half of the patients had increased cerebral perfusion (sum of RCCA and LCCA, or sum of RCCA and LSA in patients with an occluded LCCA and in patients with auxiliary LCCA–LSA bypass) of 8.7% at most, and the other four patients showed slightly decreased perfusion at 5.3% or less. The detailed data are listed in Table 2.

**Table 2:** Perfusion ratio changes of descending aorta and branches.

**Cases**	**Branches with SGs**	**DA**	**IA**	**RSA**	**RCCA**	**LCCA/(LSA-LCCA)^#^**	RCCA+LCCA/(RCCA +LSA)*
Case 1	IA & LCCA	↑ (83.3~86.4%)	↓ (9.9~8.9%)	↑	↓	↑	↓ (11.6~9.0%)
Case 2	IA & LSA	↓ (75.5~70.6%)	↑ (15.7~19.5%)	↑	↓	↑^#^	↑ (5.5~6.8%)*
Case 3	IA & LCCA	– (69.6~70.0%)	↓ (17.9~16.8%)	↓	–	↑	↑ (13.4~22.1%)
Case 4	IA & LCCA	↑ (83.8~85.4%)	↑ (8.2~9.8%)	↓	↑	↑	↑ (5.8~9.9%)
Case 5	IA & LSA	↑ (77.2~85.6%)	↓ (12.8~9.2%)	↓	↓	↑^#^	↑ (8.2~8.9%)*
Case 6	IA & LCCA	↑ (70.7~84.7%)	↓ (15.9~9.7%)	↓	↓	↓	↓ (13.9~9.4%)
Case 7	IA & LCCA	↑ (77.0~86.2%)	↓ (11.4~7.6%)	↓	↓	–	↓ (14.0~11.2%)
Case 8	IA & LSA	↑ (62.2~79.5%)	↓ (19.8~9.4%)	↓	↓	NA	↓ (21.9~16.6%)*

#, cases with LSA–LCCA bypass; ^*^, perfusion of RCCA + LSA; ↑, increased; ↓, decreased; –, unchanged; NA, not available (occluded left internal carotid artery preoperatively); DA, descending aorta; IA, innominate artery; LCCA, left common carotid artery; RCCA, right common carotid artery; LSA, left subclavian artery; RSA, right subclavian artery; SGs, stent-grafts.

**Figure 5:**
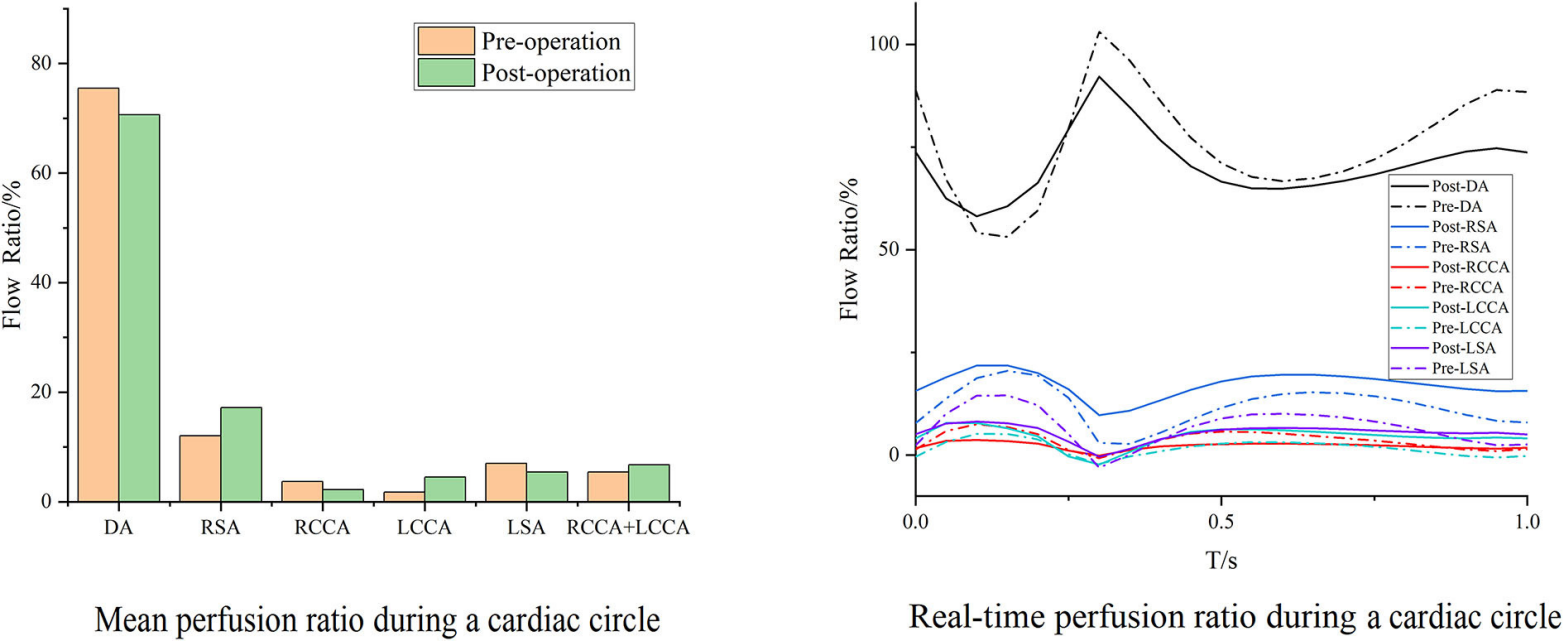
Blood flow perfusion ratios of branches in Case 2. The bar graph illustrates the changes in the flow perfusion ratios in different branches. The curve figure illustrates the real-time perfusion within a cardiac cycle. Curves below the 0 level indicate backflow. DA, descending aorta; LCCA, left common carotid artery; RCCA, right common carotid artery; LSA, left subclavian artery; RSA, right subclavian artery.

**Figure 6:**
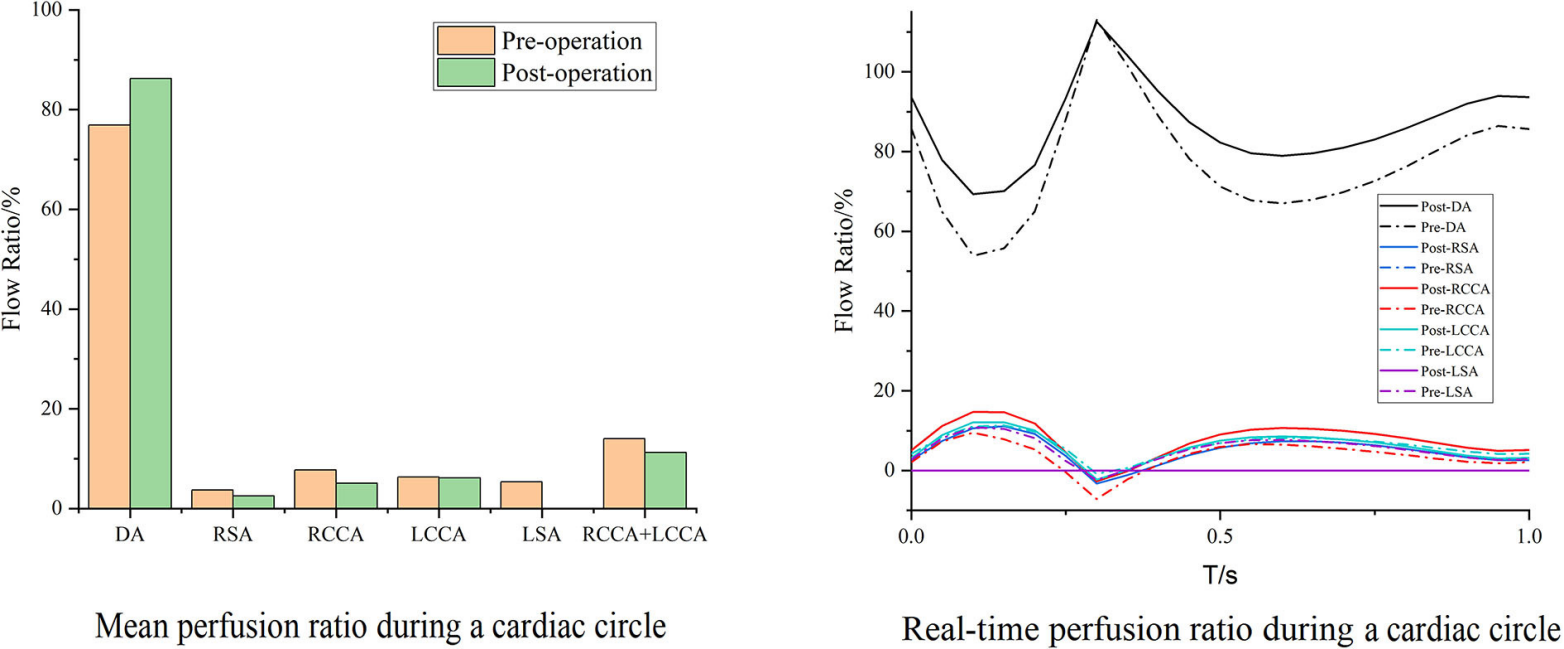
Blood flow perfusion ratios of branches in Case 7. The bar graph illustrates the changes in the flow perfusion ratios in different branches. The curve figure illustrates the real-time perfusion within a cardiac cycle. Curves below the 0 level indicate backflow. DA, descending aorta; LCCA, left common carotid artery; RCCA, right common carotid artery; LSA, left subclavian artery; RSA, right subclavian artery.

Cases 2 and 7 represented opposite trends of perfusion changes. In Case 2, the perfusion increased in all branches except the RCCA, and the cerebral perfusion (sum of RCCA and LCCA) exhibited improvement. Within the whole cardiac cycle, backflow was observed in all branches except the RCCA (Figure 5). In contrast, the branch perfusions in Case 7 generally decreased (Figure 6). The perfusion in the DA within the whole cardiac cycle obviously increased; the perfusion in the LCCA stayed roughly the same; and the perfusion in the RSA, RCCA, and cerebral perfusion (sum of RCCA and LCCA) decreased compared with the preoperative baseline. Additionally, backflow was observed in all branches before and after TEVAR.

**Figure 7:**
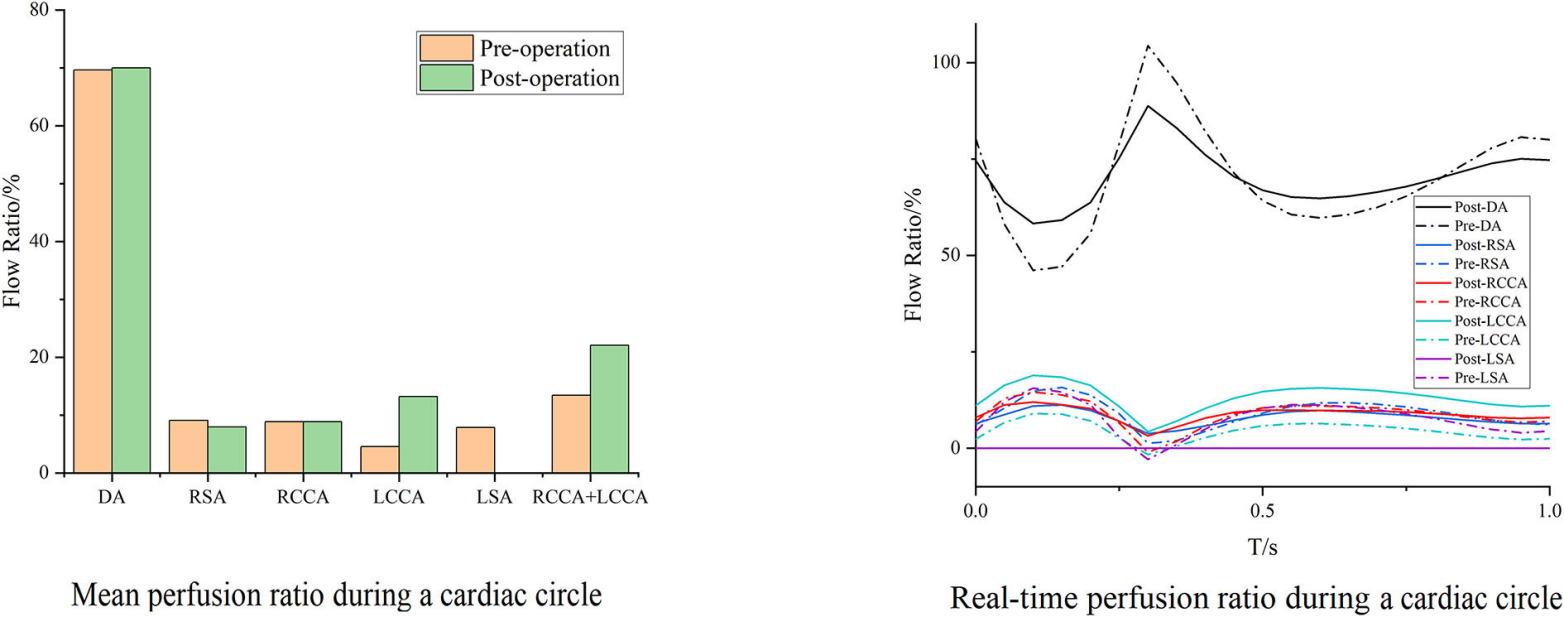
Blood flow perfusion ratios of branches in Case 3. The bar graph illustrates the changes in the flow perfusion ratios in different branches. The curve figure illustrates the real-time perfusion within a cardiac cycle. Curves below the 0 level indicate backflow. DA, descending aorta; LCCA, left common carotid artery; RCCA, right common carotid artery; LSA, left subclavian artery; RSA, right subclavian artery.

Case 3 is the unique patient with the unintended branch courses that the third module of the MIBSG coursed through the intersection angle between the two bridging SGs of the second module, leading to the separation of the bridging SGs on both sides of the third module instead of the desired morphology in the other seven cases. Despite this, the perfusions in Case 3 unexpectedly improved to a substantial degree. The perfusion of the DA changed slightly, whereas the cerebral perfusion increased. Furthermore, during a single cardiac cycle, the amplitude between the maximum and minimum flow volume decreased compared with the preoperative baseline, and no backflow was observed (Figure 7).

#### Hemodynamic indicators

The postoperative WSS increased in two cases (Cases 4 and 7). They shared the same geometric features of the existing residual profile of the aneurysm postoperatively, and their areas containing a TAWSS of <0.4 Pa increased while the values decreased in the other six cases. Areas with an OSI of >0.25, indicating greater fluctuation of blood flow, increased postoperatively for all cases except Case 6. The areas with an RRT of >5/Pa increased in three cases, with larger values inferring a higher risk of atherosclerosis or stenosis (Table 3).

**Table 3:** Area changes of hemodynamic indicators.

**Cases**	**TAWSS <0.4Pa**	**OSI>0.25**	**RRT>5(1/pa)**	**Areas increased in IA SGs**	**Areas increased in LCCA/LSA SGs**
Case 1	↓	↑	↑	OSI, RRT	OSI, RRT
Case 2^#^	↓	↑	↓	None	TAWSS, OSI, RRT
Case 3	↓	↑	↓	None	None
Case 4	↑	↑	↑	TAWSS, OSI, RRT	TAWSS, OSI, RRT
Case 5^#^	↓	↑	↓	TAWSS, OSI, RRT	None
Case 6	↓	↓	↓	TAWSS, OSI, RRT	None
Case 7	↑	↑	↑	TAWSS, OSI, RRT	TAWSS, OSI, RRT
Case 8^#^	↓	↑	↓	TAWSS, OSI, RRT	None

#, cases with LSA SGs; ↑, increased; ↓, decreased; IA, innominate artery; LCCA, left common carotid artery; LSA, left subclavian artery; OSI, oscillatory shear index; RRT, relative residence time; SGs, stent-grafts; TAWSS, time-averaged wall shear stress.

**Figure 8:**
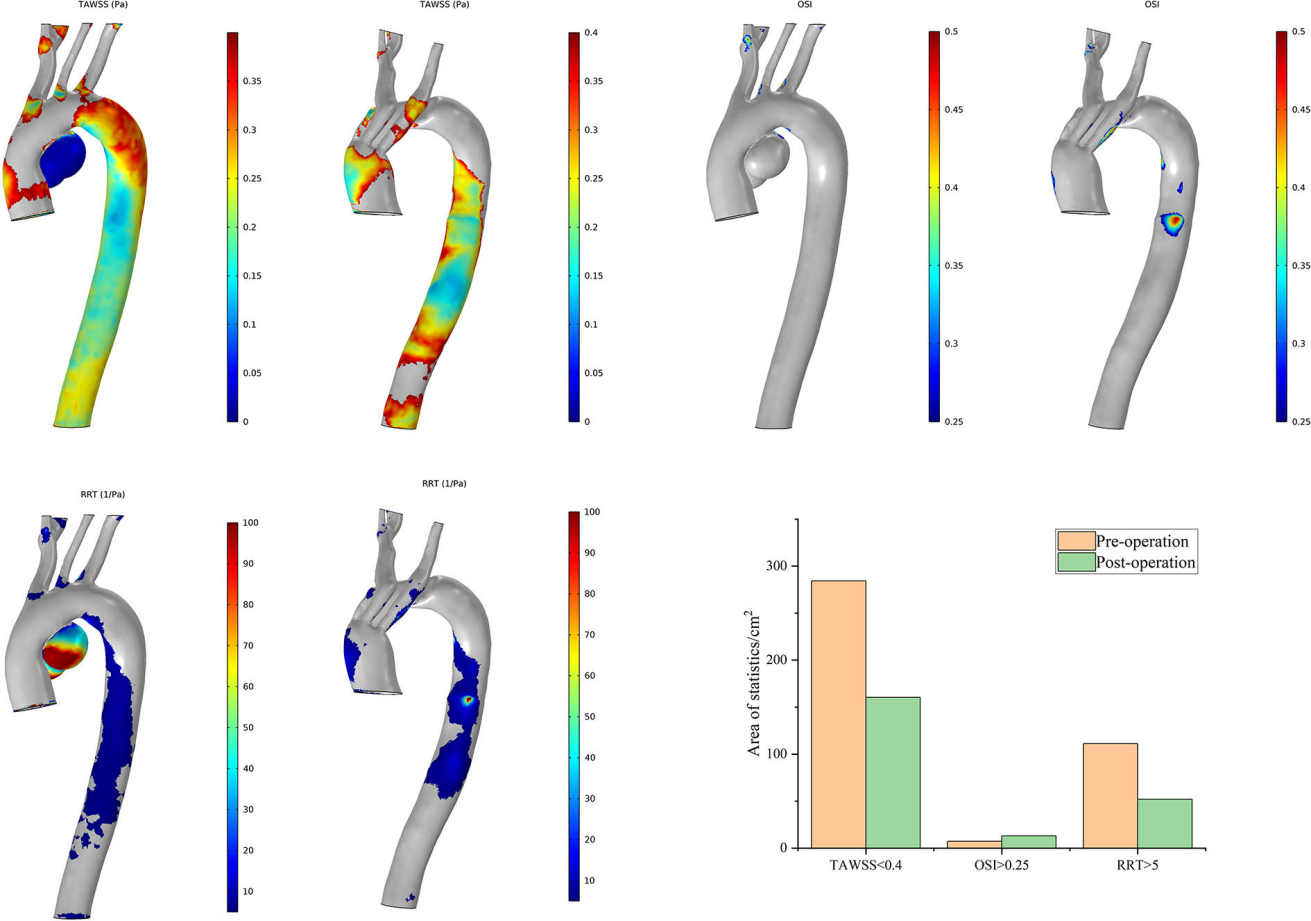
Distributions of changes in TAWSS, OSI, and RRT in Case 2. The hemodynamic characteristic regions are visually illustrated as a color distribution nephogram with each indicator in a group (left, preoperative; right, postoperative). Areas were calculated and illustrated in the bar graph. OSI, oscillatory shear index; RRT, relative residence time; TAWSS, time-averaged wall shear stress.

With the same consideration as in the aforementioned blood perfusion analyses, the hemodynamic features of Cases 2 and 7 were also analyzed in detail. The distributions of the TAWSS, OSI, and RRT in Case 2 are depicted in Figure 8. Preoperative characteristic TAWSS regions were mainly located at the aneurysm, the ostia of branches, and the DA. The total areas of a low postoperative TAWSS (<0.4 Pa) decreased significantly, while characteristic regions increased locally in the bridging SG in the LCCA. Areas with a higher OSI (>0.25) slightly increased in the LCCA and DA. The changes in the RRT were similar to the changes in the TAWSS. The preoperative flow patterns were complicated because of the aneurysm location and size. The aneurysm was sealed, after which the blood flow streamlines became smoother. The distributions of the postoperative TAWSS and RRT then significantly improved.

**Figure 9:**
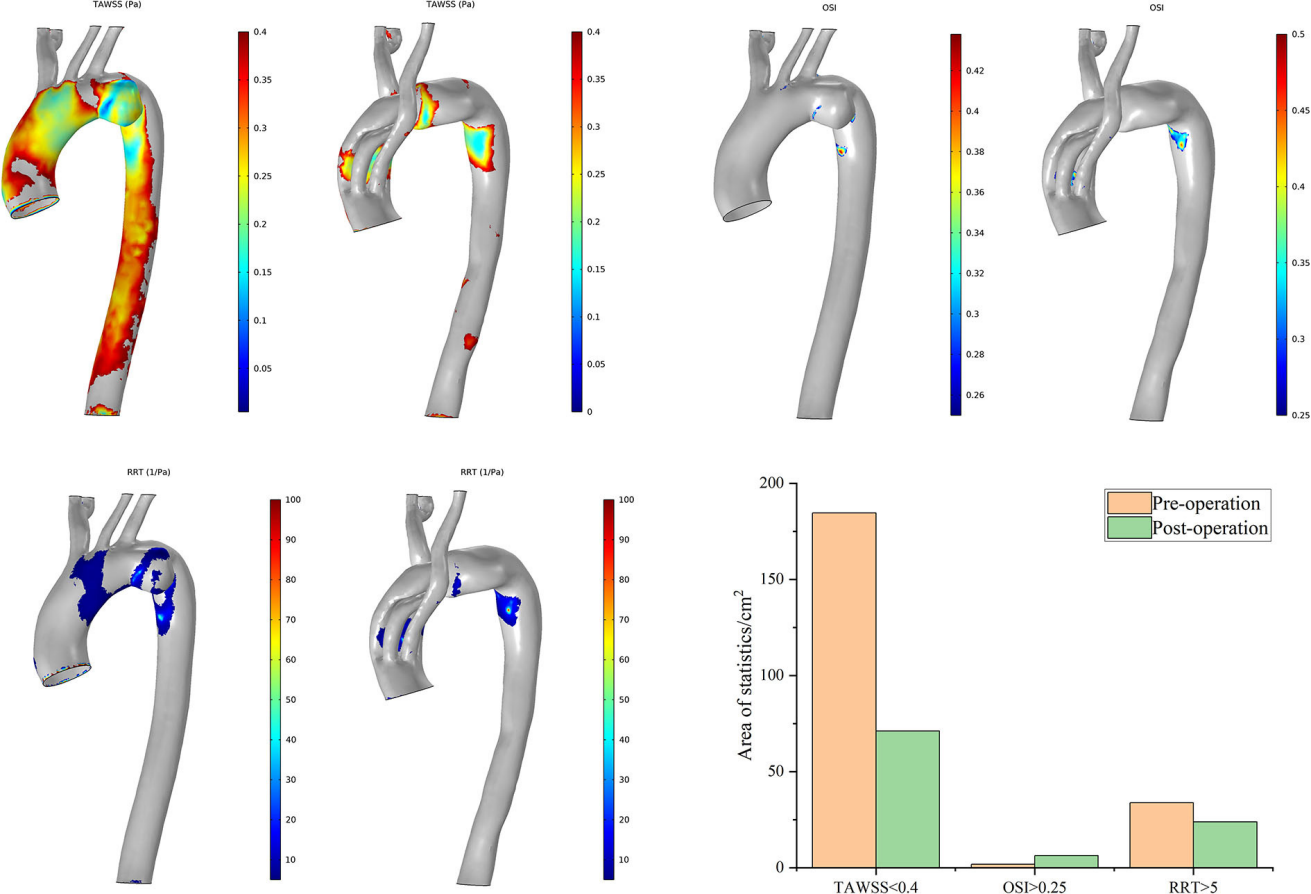
Distributions of changes in TAWSS, OSI, and RRT in Case 7. The hemodynamic characteristic regions are visually illustrated as a color distribution nephogram with each indicator in a group (left, preoperative; right, postoperative). Areas were calculated and illustrated in the bar graph. OSI, oscillatory shear index; RRT, relative residence time; TAWSS, time-averaged wall shear stress.

The postoperative characteristic regions of Case 7 increased (Figure 9). Regions with a lower TAWSS (<0.4 Pa) appeared mainly at the former aneurysm site and in the lumens of SGs in the IA and LCCA. The regions with a larger OSI (>0.25) and RRT (>5/Pa) showed similar distributions. The characteristic regions appeared in the branched SGs, indicating fluctuation of blood flow to some extent. The postoperative residual profile of the aneurysm may have been the geometric morphological cause of these flow patterns.

**Figure 10:**
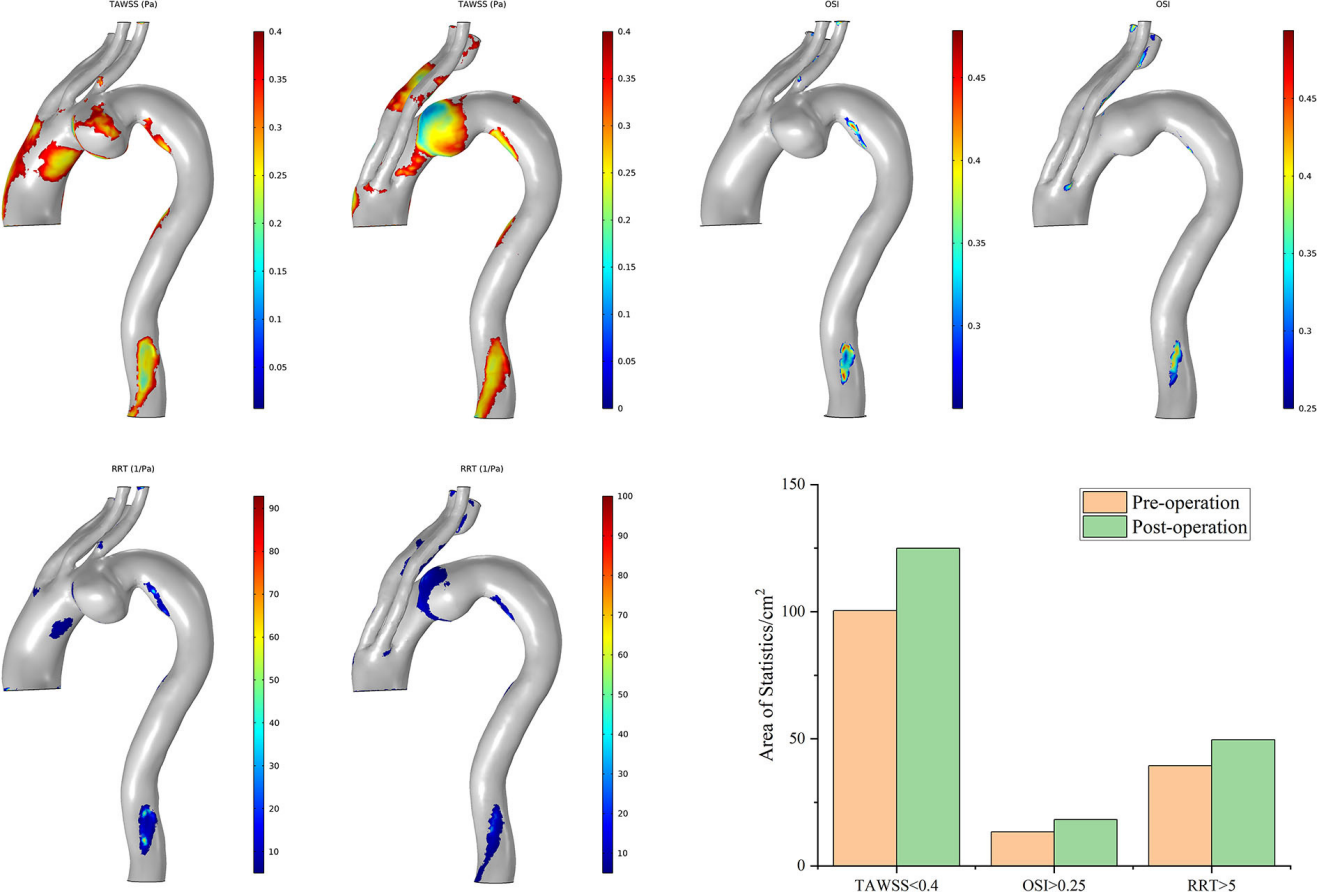
Distributions of changes in TAWSS, OSI, and RRT in Case 3. The hemodynamic characteristic regions are visually illustrated as a color distribution nephogram with each indicator in a group (left, preoperative; right, postoperative). Areas were calculated and illustrated in the bar graph. OSI, oscillatory shear index; RRT, relative residence time; TAWSS, time-averaged wall shear stress.

Case 3 has obtained the greatest improvement in hemodynamic features according to the postoperative distributions of high-risk regions (Figure 10). The preoperative high-risk regions were located mainly around the ascending aorta, the aneurysmal sac, and the inner curve of the DA. There was less distribution of characteristic regions in the branch vessels, and it remained tiny postoperatively. This feature is very different from that of other cases. The postoperative areas with a TAWSS of <0.4 Pa decreased significantly. The distribution of these regions appeared mostly around the intersection point between the second and third modules. The postoperative distribution of an RRT of >5/Pa was highly consistent with that of a TAWSS of <0.4 Pa. Regions with an OSI of >0.25 increased slightly around the overlap zone of the inner branched SGs. In general, the above-mentioned special postoperative morphology of the bridging SGs did not complicate the blood flow pattern, and ideal hemodynamic features were observed in Case 3.

## Discussion

The hemodynamic characteristics of the aortic arch are complex because of its special anatomical morphology. On this basis, it is superimposed on the influences from either the lesions or the SGs, giving local individualized fluid and solid mechanical properties. These features are different from normal physiological conditions and are closely related to the long-term outcomes of TEVAR. In recent years, scholars have attempted to analyze the hemodynamic characteristics of AAPs through numerical simulation to better understand the pathophysiological influences of intervention (20, 21). Some studies have confirmed that the pulsating blood flow is subjected to the dual effects of the radial pressure gradient and centrifugal force when it flows through a twisted arch at high speed, and secondary flow thus forms perpendicular to the main flow direction (22, 23). Furthermore, the streamlines in the supra-arch branches are twisted to the distal end of the vessels, and a reflux phenomenon occurs at the proximal end of the branches (24, 25). These characteristics were verified in our simulation under steady-state conditions. Backflows were also observed preoperatively in most branches under normal physiological conditions. This is not the unique phenomenon caused by MIBSG implantation. In this respect, there is no significant change in the postoperative flow pattern.

Minimization of neurological complications remains a major concern of all procedures addressing AAPs, irrespective of whether open surgery or TEVAR is performed. The intra- and post-operation strokes are generally due to the embolism during manipulation. While the influence of that if the long-term flow perfusion changes after MIBSG intervention increases, the risk of late neurological adverse events should be considered. Perfusion of the supra-arch branches can be evaluated *via* numerical simulation analyses. Our results showed that the perfusion increased in the DA in most cases and accordingly decreased in the brachiocephalic vessels. Interestingly, however, elevated LCCA perfusion was observed in most cases, although the RCCA perfusion showed a general reduction. As a result of this, the cerebral perfusion increased in four cases but decreased in the other four cases with acceptable variations. In this sense, the redistribution of blood flow after MIBSG deployment does not necessarily increase the risk of neurological complications. The numerical simulation showed that both the flow rate and flow resistance were associated with the vessel diameters and the winding courses of the branches. Perfusion in the RCCA could be improved by enlarging the inner-branch diameters and adjusting the direction of the inner-branch tunnels. The parameters of the MIBSG could be accordingly modified to lower the flow resistance and reduce the tortuosity of the bridging SGs, eventually obtaining optimized hemodynamic properties.

From a hemodynamic viewpoint, regions with a TAWSS of <0.4 Pa, OSI of >0.25, and RRT of >5/Pa indicate a higher risk of atherosclerosis. In six cases, the improvements in low blood flow mechanical parameters were identified for that the areas with a TAWSS of <0.4 Pa decreased. A higher RRT indicates a longer time during which tangible substances stay in the vessels. Thus, the distribution of high-RRT regions is helpful to locate high-risk sites in which deposits are likely to form, eventually leading to atherosclerosis or restenosis. High-RRT areas increased in three cases in this study, and further follow-up will be required in these cases. A higher OSI represents a greater oscillation intensity in the shear stress direction and more frequent changes in the flow direction. In this study, areas with an OSI of >0.25 increased in most cases. This indicates that the disorder of blood flow characteristics increased throughout the whole cardiac cycle, resulting in the oscillation of the flow direction in the SGs. We inferred that this was related to the fact that the bridging SGs lengthened the distance of the supra-arch trunks from the top of the aortic arch into the proximal ascending aorta. Changes in the diameters and directions along this route can lead to streamline disorder. This is thought to be a systematic risk that originates from the unique structure of the devices used and cannot be completely avoided. To some extent, it is a limitation of all TEVAR procedures.

In general, the postoperative hemodynamic properties remarkably improved in most cases as indicated by the fact that the preoperative characteristic regions (which were mainly located around the aneurysm sac, the ostia or bifurcations of branches, and the DA) significantly decreased after coverage with the MIBSGs. However, the postoperative characteristic regions appeared in the lumens of the branched SGs in some cases (e.g., Case 7). Although the impact on the vessel intima may be relatively low because of the protection provided by the SG membrane, there are still long-term risks of atherosclerosis and subsequent restenosis in patients with postoperative residual characteristic regions around either the interface between the SGs and vessels or the IA bifurcation. Therefore, close follow-up is needed. Notably, the greatest hemodynamic improvement was observed in Case 3, which involved the unexpected deployment of the third module crossing the intersection angle between the two inner-branch SGs. This is inconsistent with the existing understanding based on CTA morphology. The presumed reason may be that the axes of both the first and second modules were almost parallel with the axis of the ascending aorta. Thus, there was minimal impact on the streamlines in this region.

These hemodynamic analysis results are helpful for predicting the risks of an adverse prognosis in individual patients. They can also be utilized in preoperative planning. For a patient who is presumed to have poor postoperative hemodynamic characteristics, the clinician may re-examine the rationality of the operative plan or individualize the devices to improve their hemodynamic performance. We recommend this hemodynamic evaluation as an important criterion for preoperative planning and postoperative follow-up in patients undergoing TEVAR involving the aortic arch.

### Limitations

This study had two main limitations. First, to simplify the complex calculations, the rigidity of the implants was not considered. Second, the number of cases was small and the follow-up time was relatively short. The long-term result is needed to prove the effect of hemodynamic numerical simulation.

## Conclusion

The MIBSG device provided promising early-term results in these eight first-in-human cases. The numerical analysis showed improved hemodynamic features of the aorta and supra-arch branches in most cases. Some patients required close follow-up because of the increased hemodynamic risks of late complications. The design of the MIBSG could be partly modified to acquire better hemodynamic performance and thus improve the long-term outcomes. Hemodynamic numerical simulation is a clinically valuable way to guide TEVAR management. Additional evidence is needed in patients with aortic arch involvement.

## Data availability statement

The raw data supporting the conclusions of this article will be made available by the authors, without undue reservation.

## Ethics statement

The studies involving human participants were reviewed and approved by Ethics Committee of the Chinese People's Liberation Army General Hospital (S2018-230-01). The patients/participants provided their written informed consent to participate in this study. Written informed consent was obtained from the individual(s) for the publication of any potentially identifiable images or data included in this article.

## Author contributions

WG, YZ, FL, and HZ designed the study. YZ, FL, HS, XM, and WZ analyzed and interpreted the data. HZ and LC collected the data. YZ and FL drafted the article. All authors listed have made a substantial, direct, and intellectual contribution to the work and approved it for publication.

## Funding

This research was supported by the Beijing Municipal Natural Science Foundation (Grant Number 7212095) and Capital's Funds for Health Improvement and Research (Grant Number 2020-2Z-5014).

## Conflict of interest

WG is the designer of the modular inner branched stent-graft and the principal investigator of the GIANT clinical trials. The remaining authors declare that the research was conducted in the absence of any commercial or financial relationships that could be construed as a potential conflict of interest.

## Publisher's note

All claims expressed in this article are solely those of the authors and do not necessarily represent those of their affiliated organizations, or those of the publisher, the editors and the reviewers. Any product that may be evaluated in this article, or claim that may be made by its manufacturer, is not guaranteed or endorsed by the publisher.

## Abbreviations

AAPs: aortic arch pathologies
CTA: computed tomography angiography
DA: descending aorta
IA: innominate artery
LCCA: left common carotid artery
RCCA: right common carotid artery
LSA: left subclavian artery
RSA: right subclavian artery
MIBSG: modular inner branched stent-graft
OSI: oscillatory shear index
RRT: relative residence time
SGs: stent-grafts
TAWSS: time-averaged wall shear stress
TEVAR: thoracic endovascular aortic repair
WSS: wall shear stress.
